# Design of a two functional permeable reactive barrier for synergistic enzymatic and microbial bioremediation of phenol-contaminated waters: laboratory column evaluation: Enzymatic and microbial bioremediation of phenol in a bilayer permeable reactive barrier

**DOI:** 10.1186/s12866-024-03413-2

**Published:** 2024-07-09

**Authors:** Sayed Hossein Mirdamadian, Sedigheh Asad, Seyed Mohammad Mehdi Dastgheib, Hamid Moghimi

**Affiliations:** 1 Department of Microbiology, School of Biology, College of Science, University of Tehran, Tehran, Iran; 2 Department of Biotechnology, College of Science, University of Tehran, Tehran, Iran; 3 Microbiology and Biotechnology Research Group, Research Institute of Petroleum Industry, Tehran, Iran

**Keywords:** Bioremediation, Horseradish peroxidase, Microbial degradation, Permeable reactive barrier, Phenol

## Abstract

The present study aimed to develop a system using a combination of enzymatic and microbial degradation techniques for removing phenol from contaminated water. In our prior research, the HRP enzyme extracted from horseradish roots was utilized within a core-shell microcapsule to reduce phenolic shock, serving as a monolayer column. To complete the phenol removal process, a second column containing degrading microorganisms was added to the last column in this research. Phenol-degrading bacteria were isolated from different microbial sources on a phenolic base medium. Additionally, encapsulated calcium peroxide nanoparticles were used to provide dissolved oxygen for the microbial population. Results showed that the both isolated strains, WC1 and CC1, were able to completely remove phenol from the contaminated influent water the range within 5 to 7 days, respectively. Molecular identification showed 99.8% similarity for WC1 isolate to *Stenotrophomonas rizophila* strain e-p10 and 99.9% similarity for CC1 isolate to *Bacillus cereus* strain IAM 12,605. The results also indicated that columns using activated sludge as a microbial source had the highest removal rate, with the microbial biofilm completely removing 100% of the 100 mg/L phenol concentration in contaminated influent water after 40 days. Finally, the concurrent use of core-shell microcapsules containing enzymes and capsules containing *Stenotrophomonas* sp. WC1 strain in two continuous column reactors was able to completely remove phenol from polluted water with a concentration of 500 mg/L for a period of 20 days. The results suggest that a combination of enzymatic and microbial degrading systems can be used as a new system to remove phenol from polluted streams with higher concentrations of phenol by eliminating the shock of phenol on the microbial population.

## Introduction

Phenol and its derivatives usually exist in wastewater produced by industries that use phenol as the main component of their products. Phenol at concentrations greater than five milligrams per liter can be harmful to human health; while the presence of phenol in water and wastewater at concentrations greater than one milligram per liter can also have adverse effects on aquatic organisms in these environments [1, 2].

Groundwater and purified wastewater are the main sources of water in the world and have an essential contribution to the life of living organisms and human societies. One of the proposed mechanisms for the remediation of polluted groundwaters and wastewaters is the use of biological treatment methods by permeable reactive barriers (PRBs). PRBs are an effective and environmentally friendly solution for the remediation of polluted groundwater and wastewater streams. The technology involves installing a reactive material as a barrier in the path of contaminated water, which allows for the separation and removal of pollutants through various physical, chemical, and biological processes [3, 4].

While physicochemical methods have been widely used to remove phenol from wastewater, they are not always efficient in completely removing all traces of phenol. Additionally, these methods can be energy intensive and costly [5]. In contrast, biological treatment techniques provide a more environmentally friendly and economically viable strategy to eliminate phenol from wastewater. The selection of the appropriate treatment approach relies on several factors, including the phenol concentration in the wastewater, the quantity of sewage requiring treatment, the accessibility of resources, and the prevailing environmental regulations [5, 6].

Biological techniques can be categorized into two subgroups: the enzyme treatment approach and the biological decomposition method involving microorganisms and plants. Microbial decomposition is an important and efficient strategy for removing organic compound pollutants and detoxifying wastewater [6, 7]. Phenol is degraded by a wide variety of microorganisms, including yeasts, molds, and bacteria, such as *Pseudomonas putida*,* Bacillus subtilis*,* Alcaligenes faecalis*,* Rhodococcus erythropolis* and *Corynebacterium sp.* [8]. Since the concentration of oxygen in polluted water and wastewater, especially groundwater, is low, it can limit the activity of aerobic microorganisms, so oxygen-releasing compounds (ORCs) can be used to solve this problem. The ORC represents a distinctive formulation of calcium, magnesium peroxide, that slowly break down and emit hydrogen peroxide and oxygen when exposed to water. These compounds are insoluble, but when added to water, they convert to magnesium hydroxide or calcium hydroxide and subsequently release hydrogen peroxide and molecular oxygen. The hydrogen peroxide and oxygen released during this reaction can help to increase the dissolved oxygen levels in the water and promote the growth and activity of aerobic bacteria [9, 10]. Another main problem for microbial bioremediation of phenol-contaminated waters is the prevention of microbial activities by phenol concentrations higher than 100 mg/L, which inhibits the growth of microorganisms. Enzymatic pretreatment is a helpful method to reduce the concentration of phenol in polluted wastewaters before microbial biodegradation [11, 12].

Enzymes such as peroxidases and laccases are effective in removing pollutants such as phenol from the environment. The most widely used peroxidase to remove and eliminate phenolic compounds is horseradish peroxidase (HRP, EC: 1.11.1.7). One advantage of using enzymes is their specificity, which allows them to target specific pollutants [13]. Nevertheless, there are certain difficulties linked to the utilization of complimentary enzymes for the elimination of pollutants. These challenges include inadequate stability and restricted availability of essential reactants like hydrogen peroxide. Enzyme stability and activity can be enhanced by immobilizing enzymes on a solid support. Furthermore, immobilized oxygen-releasing compounds can also serve as a hydrogen peroxide source for enzyme-catalyzed reactions [14, 15]. In our previous research, we designed a column of core-shell microcapsules containing HRP enzyme, which could remove phenol from the polluted water stream with high efficiency and maintained an efficiency of up to 50% after 15 days of operation. To complete the phenol removal process, a second column containing immobilized degrading bacteria in the presence of immobilized CaO_2_ nanoparticles as ORC was added to the previous column in this research.

The objective of this investigation was to employ immobilized HRP in the initial column with the purpose of diminishing the level of phenol in polluted water. This reduction in phenol concentration would aid in averting phenolic shock during subsequent microbial treatment procedures. Subsequently, the isolated degrading strains would be utilized in the second column to entirely eliminate phenol from the contaminated wastewater. This research is the first use of synergistic enzyme and microbial decomposition effects along with ORC nanoparticles to purify phenolic-polluted water and it is the most important innovation of this research.

## Materials and methods

### Chemicals

H_2_O_2_ (30%), phenol (99%), potassium ferricyanide, and sodium alginate were prepared from Merck (Germany). Horseradish peroxidase (HRP, Type VI, P8375, EC 1.11.1.7) and 4-aminoantipyrine (AAP) were purchased from Sigma–Aldrich Company. R2A medium was obtained from Himedia Company (India). All remaining chemical compounds were of analytical grade and obtained from internal commercial sources. Calcium peroxide nanoparticles were delivered from Dr. Mosmeri as a generous gift.

### Sample collection and source of microorganisms

The sand and groundwater used in the study were obtained from a well located near the Isfahan Refinery in Iran (32.7860455701198 N, 51.510171656097896 E). The sand underwent a meticulous preparation process involving acid washing and autoclaving to eradicate any possible instances of chemical or microbial contamination.

Two different samples, including an activated sludge and a compost sample as mixed microbial cultures, were collected from the aeration pool of the North Isfahan Wastewater Treatment Plant (32.74958227304224 N, 51.736378614698644 E) and the Isfahan Municipal Waste Organization Factory (32.61298613418583 N, 51.809297190184736 E), respectively. The microbial biofilms formed from the above samples were further used for the isolation of phenol-degrading microorganisms after column experiments.

### Encapsulation of calcium peroxide nanoparticles as ORCs

In our prior study, it was determined that CaO_2_ exhibits a greater capacity for the release of H_2_O_2_ and molecular oxygen compared to MgO_2_. Therefore, in this research, CaO_2_ was used as the ORC to supply hydrogen peroxide and molecular oxygen. To prepare the ORC beads, 0.1 g of calcium peroxide nanoparticles was added to 10 ml of distilled water, and the mixture was stirred for 5 min on a magnetic stirrer or sonic bath. The resulting mixture was then placed on a heater at a temperature of 70 °C until the temperature gradually reached 70 °C. Next, 0.1 g of sodium alginate was slowly added to the mixture, and stirring was continued until a milky and uniform jelly mixture was obtained. Afterward, the suspension was gradually dropped into a laboratory beaker containing a calcium chloride solution (1 M) using a peristaltic pump. Stirring of the mixture was continued for 30 min after the last drop was dispensed. Finally, the obtained capsules were kept at 4 °C until use [16, 17].

### Monolayer column experiment with different microbial sources

A monolayer column reactor system was employed to assess the efficacy of various microbial sources in eliminating phenol from a continuous flow of phenol-laden groundwater. Four sand-packed columns (1–3 mm diameter) were prepared to simulate the groundwater substrate, labelled R1-R4, each containing a different microbial source. In R1, the microbial flora from the groundwater was used as the microbial source, while activated sludge and compost were used in R2 and R3, respectively. All three columns were supplemented with ORC beads to generate dissolved oxygen and motivate the microbial flora. The fourth column, R4, served as a control and contained only the microbial flora from the groundwater without ORC beads. The contaminated groundwater was then passed through the column reactors, and the remaining phenol, microbial population number, DO concentration, and pH changes were measured in the effluent of the columns over a 40-day duration.

Furthermore, a parallel experimental arrangement was employed to examine the abiotic conditions and distinguish the impact of microorganisms on contaminant remediation by using sodium azide. The SPC method was used to investigate the microbial population on R2A agar medium using decimal dilution of the samples in sterile physiological serum. All experiments were directed at a temperature of 20 °C [18, 19].

### Phenol concentration determination

Phenol concentration measurement is performed by phenol activation with 4-aminoantipyrine. The remaining phenol was assessed in the effluent of reactors. The concentration of phenol is evaluated by a colorimetric reaction based on the condensation of phenol with 4-aminoantipyrine in the presence of alkalic oxidizing reagents such as K_3_FeCN_6_ (potassium ferrocyanide) for antipyrine dye formation. The formation of this dye was measured at 510 nm colorimetrically as an assay of phenol concentration described in our previous work [16, 20].

### Screening and isolation of phenol-degrading bacteria

Enrichment of phenol-degrading bacteria from collected samples and isolation from formed microbial biofilms in monolayer column experiments were used for screening. Mineral salt media (MSM) with phenol as the sole source of carbon and tryptic soy agar (TSA) were used in the present study. The constituents of the MSM medium are as follows (per litter): 0.4 g KH_2_PO_4_, 0.4 g K_2_HPO_4_, 0.2 g MgSO_4_·H_2_O, 0.1 g NaCl, 0.01 g Fe_2_(SO_4_)_3_·H_2_O, 0.01 g MnSO_4_·H_2_O, 1.0 g (NH_4_)_2_SO_4_, and 0.01 g Na_2_MoO_4_·2H_2_O. The pH of the medium culture was adjusted to 6.8. A process of enrichment culture was carried out to isolate and select phenol-degrading bacteria. The samples were inoculated into laboratory flasks containing MSM (minimal salt media) supplemented with phenol as the sole carbon source at varying concentrations (100, 200, 300, 400, 500 mg/L). The enriched culture with more biomass was further transferred into freshly prepared enrichment media containing higher phenol concentrations. After several enrichment cycles, the final enriched media was diluted serially and spread onto TSA plates supplemented with phenol. These plates were then incubated at 20 °C, and single colonies with morphological differences were selected and streaked on new plates. This process allowed for the isolation and selection of different types of phenol-degrading bacteria [21, 22].

Furthermore, bacteria capable of degrading phenol were also obtained from biofilms that developed in a monolayer column experiment. To isolate these bacteria, the effluent was evenly distributed onto plates that were cultured using MSM Agar supplemented with phenol, following a series of dilutions. The obtained bacterial isolates were subsequently preserved at a temperature of 4 °C for future investigation [23, 24].

### Identification by 16 S rDNA and phylogenetic analysis

Identification of selected strains through amplification of the 16 S rDNA gene was performed by PCR using a pair of forward (5′-AACTGGAGGAAGGTGGGGAT-3′) and reverse (5′-AGGAGGTGATCCAACCGCA-3′) primers after genomic DNA extraction. The 16 S rDNA gene was sequenced by Pishgam Company (Tehran, Iran). The resulting sequences were compared to known sequences in the NCBI database using BLAST. The evolutionary relationships among the different strains were then inferred using the maximum likelihood method and represented as a phylogenetic tree using MEGA 7.0 software. The analysis involved 16 S rDNA nucleotide sequences, and evolutionary distances were computed [24].

### Immobilization of bacterial cells in chitosan beads

The selected strains were harvested after overnight growth from 1 L of culture medium. The centrifugation process was employed at a speed of 5000 revolutions per minute for a duration of 10 min to acquire the cell pellet. Following this, the pellet was then reconstituted in 10 millilitres of phosphate buffered saline (PBS) at a density of 0.5 McFarland. To prepare a solution of chitosan, a stock containing 10% (w/v) chitosan in 0.1 M acetic acid was created in MSM medium without the presence of phenol. This chitosan solution was subsequently sterilized to ensure its purity and integrity. The bacterial cell suspension was added to 50 ml of sterilized chitosan solution and mixed by stirring on a magnetic stirrer. This chitosan cell mixture was extruded drop by drop into a cold sterile 0.4 w/v sodium tripolyphosphate (Na-TPP) solution using a peristaltic pump. The drops of chitosan cell solution were gelled to form a uniform and defined sphere upon contact with the Na-TPP solution. The immobilized beads were kept at room temperature for one hour to complete gel formation. The beads were then rinsed with MSM and distilled water to remove residual Na-TPP. The final product was chitosan beads containing immobilized bacterial cells, which could be used for various experimental purposes. Blank beads without bacterial cells were also prepared as control experiments.

### Preparation of enzymatic core and shell microcapsules

In our previous study, was detailed the process of immobilizing the HRP enzyme within an alginate shell surrounding a core filled with calcium peroxide nanoparticles [16].

### Phenol removal in bilayer column experiments

**Fig. 1:**
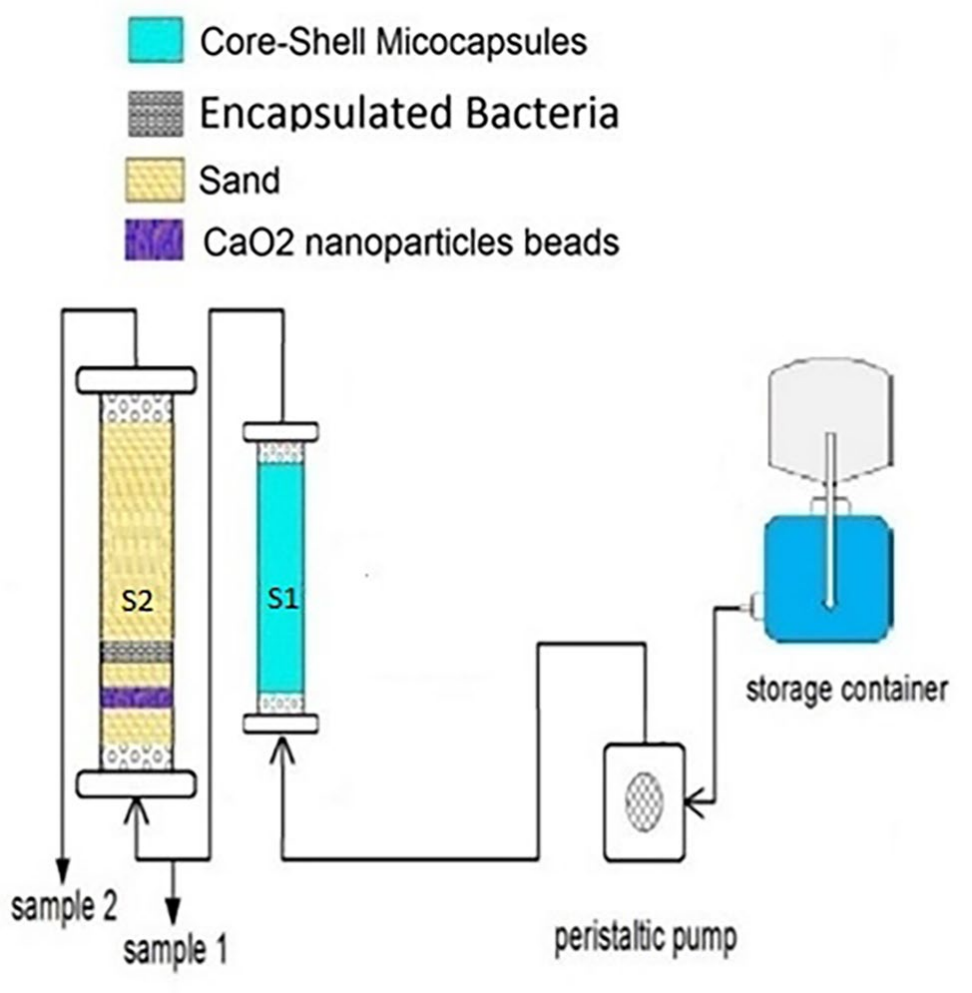
Continuous flow of contaminated water in enzymatic and microbial column reactors sequentially

The design of a dual-column sequential system was developed to effectively eliminate phenol from polluted water sources through a synergistic approach. The first column, referred to as S1, was a packed bed bioreactor with a 20 cm height and an inner radius of 2 cm, filled with 250 core-shell microcapsules containing crude enzyme extract. The 250 ml graduated cylindrical column, known as S2, was filled with washed and sterilized sand. Additionally, it contained capsules that housed the isolated microbial strains and ORC capsules. To test the system’s efficiency, synthetic phenol-contaminated water with a concentration of 500 mg/L was continuously passed through the columns at a rate of approximately 200 ml per day using a peristaltic pump. The contaminated water flowed through S1 first and then S2 in a sequential manner (Fig. 1). The goal was to achieve maximum phenol removal from the water flow over 30 days. After running the system for 30 days, the amount of phenol removal from the water flow was measured. The sequential system showed promising results, indicating efficient phenol removal from contaminated water [25, 26].

### Data analysis

The average results (± standard deviation) from at least three independent experiments were studied and analysed using IBM SPSS software (version 27.0). The Kolmogorov‒Smirnov test was utilized to assess the distribution normality of the investigated parameters, while Tukey’s HSD test was conducted to ascertain the presence of significant mean variances among the groups. Differences were considered significant at *p* < 0.05. It should be noted that all data in this research were normally distributed.

## Results and discussion

### Monolayer column experiment

**Fig. 2:**
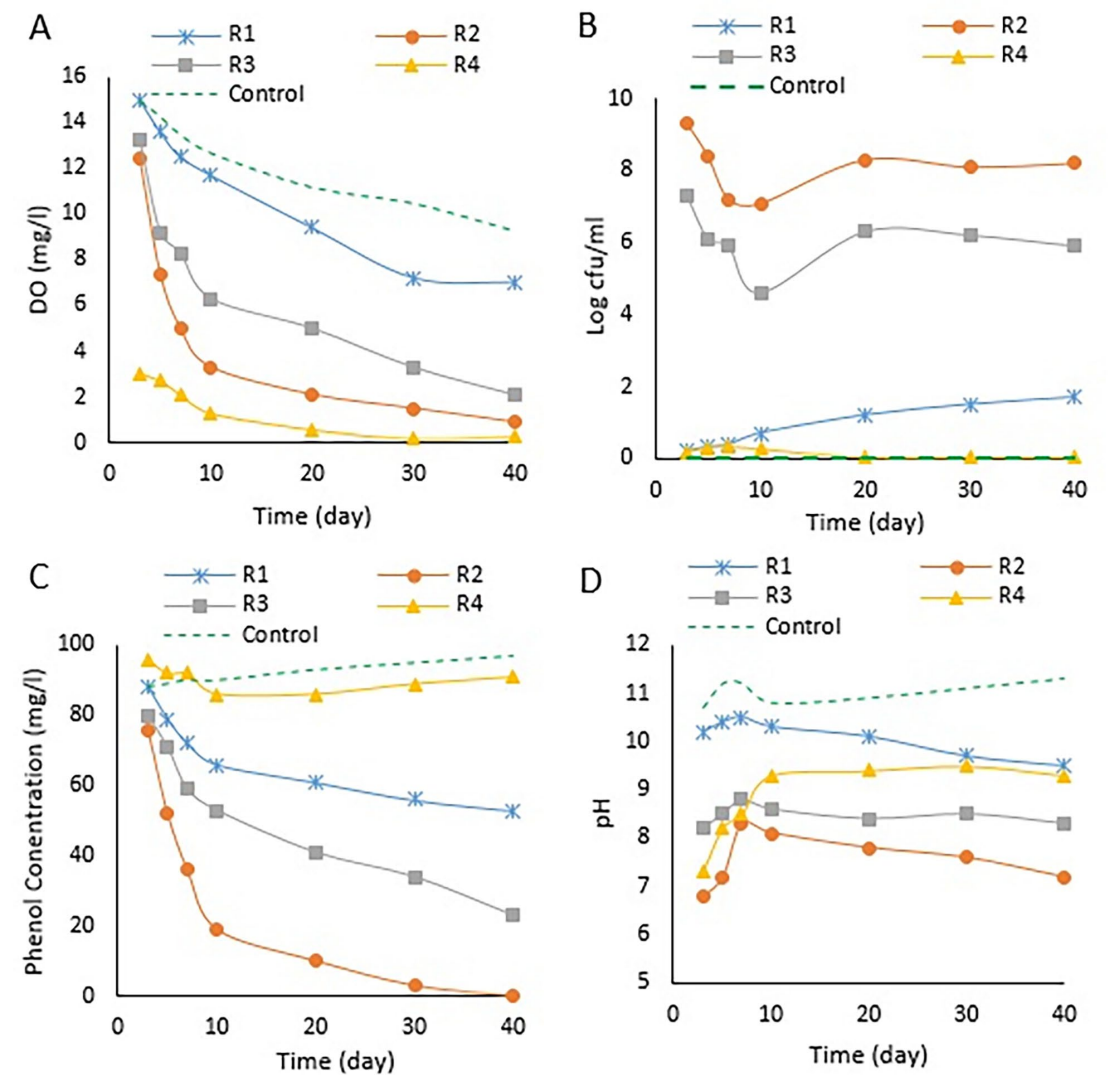
The graph displays the results obtained from an investigation into the performance of ORC capsules and different microbial sources in continuous flow columns of water contaminated with phenol at a concentration of 100 mg/litter. The study measured changes in four parameters: (**A**) dissolved oxygen concentration, (**B**) microbial population, (**C**) residual phenol concentration in outlet water, and (**D**) pH levels. The presented data show the average of three replicate samples

Four laboratory columns were employed in this study to investigate the efficacy of various microbial sources in the treatment of phenol-contaminated water. The columns were operated for 40 days with a retention time of one day, and the variations in microbial population, pH, and dissolved oxygen in the effluent were monitored. Figure 2 shows the results of these monitoring efforts.

#### Variation in dissolved oxygen concentration

Based on the results presented in Fig. 2-A, it can be concluded that the presence of ORC capsules in the effluent samples increased the concentration of dissolved oxygen in all columns, especially during the first few days of the system’s setup. However, the DO concentration gradually decreased over time in all columns, including the sodium azide-treated column, which had no microbial activity. The decline in dissolved oxygen concentration in columns R1 to R3 was mainly attributed to the actions of the microbial community and the consumption of oxygen. On the other hand, in the sodium azide column, the decrease in DO concentration was likely due to the dissociation of calcium peroxide nanoparticles in the ORC capsules. Column R4, which lacked ORC capsules, had an initial DO concentration of approximately 3 mg/L and gradually reached zero with the relative activity of the microbial population. In general, the findings indicate that the inclusion of ORC capsules has the potential to enhance the levels of dissolved oxygen in wastewater treatment systems. However, it is important to note that this effect may gradually decrease over a period of time. It seems that the addition of calcium peroxide (CaO_2_) capsules to the remediation setup resulted in an increase in the dissolved oxygen (DO) concentration in the columns, which is consistent with the findings of Gholami et al. However, it appears that in biotic conditions, the oxygen level of the groundwater dropped sharply due to O_2_ consumption by the microbial community [27]. This implies that the existence of microorganisms can exert a substantial influence on the oxygen concentrations in groundwater and must be considered when designing remediation strategies.

#### Variation in microbial population

The provided information suggests that in the effluent from columns R2 and R3, the microbial population initially decreased but then increased exponentially and remained stable (Fig. 2-B). However, there were no significant changes in the microbial population of columns R4 and R1, which may be due to the absence of an ORC source. The control column showed no microbial population due to sodium azide. The research conducted by Mosmeri and colleagues has demonstrated that elevating the levels of dissolved oxygen in groundwater can result in a rise in microbial proliferation and activity. However, this heightened activity is observed to diminish after a period of 50 days as a consequence of insufficient oxygen supply for the microorganisms. Additionally, in the research conducted by Yah et al., it was shown that when the microbial population is exposed to BTEX, it decreases first due to shock loading and then increases by adapting to the existing conditions [18, 28].

#### Variation in phenol concentration

Figure 2-C describes the results of a study on removing phenol from effluent in different columns. The study found that the microbial population in the R2 and R3 columns increased during the process, leading to more significant phenol reduction in these columns. Moreover, the results showed that the sludge microbial population in column R2 was more effective in removing phenol than that in column R3. In the column containing sodium azide, although there was no microbial activity observed, approximately 12% of the phenol that entered was eliminated through absorption by the sand bed and ORC capsules. However, the destruction of these capsules decreased this absorption capacity. In column R4, which did not use ORC capsules, only 4% of the input phenol was removed. Previous studies have reported removing different aromatic compounds, such as benzene, naphthalene, and toluene, in permeable reactive barrier systems in the presence of oxygen-releasing compounds [17, 29]. However, this study is the first report on the removal of phenol in the presence of ORCs in such systems.

#### Variation in pH

Based on the information provided, it can be inferred that the pH changes observed in the effluent of the different columns during the process were due to the alkaline effect of calcium peroxide nanoparticles and the presence of phenol (Fig. 2-D). The control column, comprising of nanoparticles of calcium peroxide, exhibited a fluctuation in pH ranging from 10 to 11. In contrast, the R4 column, which did not contain calcium peroxide nanoparticles but had phenol, showed a lower pH variation between 8 and 9. The pH value in the R2 and R3 columns was initially neutral and gradually increased. However, these pH changes caused adverse effects on the microbial population, leading to a decrease in the first few days of operation. This result was consistent with the findings of Zhou et al., who used CaO_2_ during glucose degradation and nitrification. They showed that the pH maintained alkalinity throughout the experiment by adding CaO_2_ powder [30].

### Screening and isolation of phenol-degrading bacteria from monolayer column effluents

Based on this study, the enrichment method was utilized to isolate specific bacteria from a diverse natural population. Six different bacterial strains were selected, but the WC1 and CC1 strains demonstrated better growth in phenol broth medium. These strains were isolated from sludge and compost columns (R2 and R3), respectively. These isolates were further checked for their ability to grow on MSM broth medium with different phenol concentrations. The study found that the growth of these strains was affected by higher concentrations of phenol, suggesting their sensitivity towards phenol (500 mg/L). Lowery et al. discovered a soil bacterium capable of utilizing phenol as its sole carbon and energy source. This strain was efficient in degrading phenol completely, but only when the initial concentration was less than 600 mg L − 1. This sensitivity towards higher phenol concentrations suggests that the bacteria may require acclimatization before they can fully degrade phenol.

**Fig. 3:**
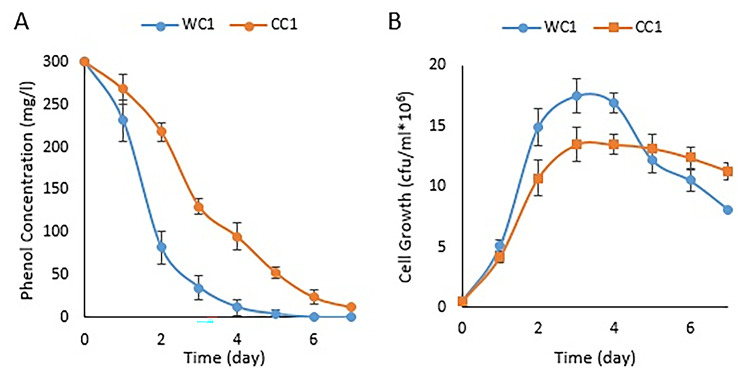
Graph of the residual phenol concentration (**A**) and the growth rate (**B**) in phenol broth medium with a concentration of 300 mg/L phenol by the strains isolated from the columns in one week. The results shown are from three replicates

In this research, all six strains degraded phenol completely for an initial concentration of 300 mg/L within seven days. However, it was observed that strains WC1 and CC1 exhibited different levels of phenol-degrading activity, with WC1 showing the highest degradation rate and CC1 exhibiting lower activity (Fig. 3A-B). Interestingly, both the growth of these strains and their ability to degrade phenol appeared to be correlated, with no lag phase observed and rapid growth occurring immediately upon inoculation. The rapid growth phase lasted for 3 days, when the phenol concentration decreased by approximately 50%. However, after the fourth day, cell growth was reduced considerably. It is interesting to note that higher phenol concentrations had a negative effect on the growth of the bacterium. This could be due to several factors, such as toxicity or inhibitory effects of accumulated metabolic by-products [31]. However, Nouri et al. reported a strain of *Trichosporon cutaneum* that can remove phenol with an efficiency of 99% at a concentration of 1250 mg/L [32].

These findings provide valuable insights into the potential use of bacterial strains in bioremediation efforts, as they suggest that certain strains may be more effective at breaking down environmental contaminants such as phenol than others. Further research may be needed to investigate the specific mechanisms underlying these differences in phenol-degrading activity and growth dynamics among the different tested strains.

### Molecular identification of phenol-degrading isolates

**Fig. 4:**
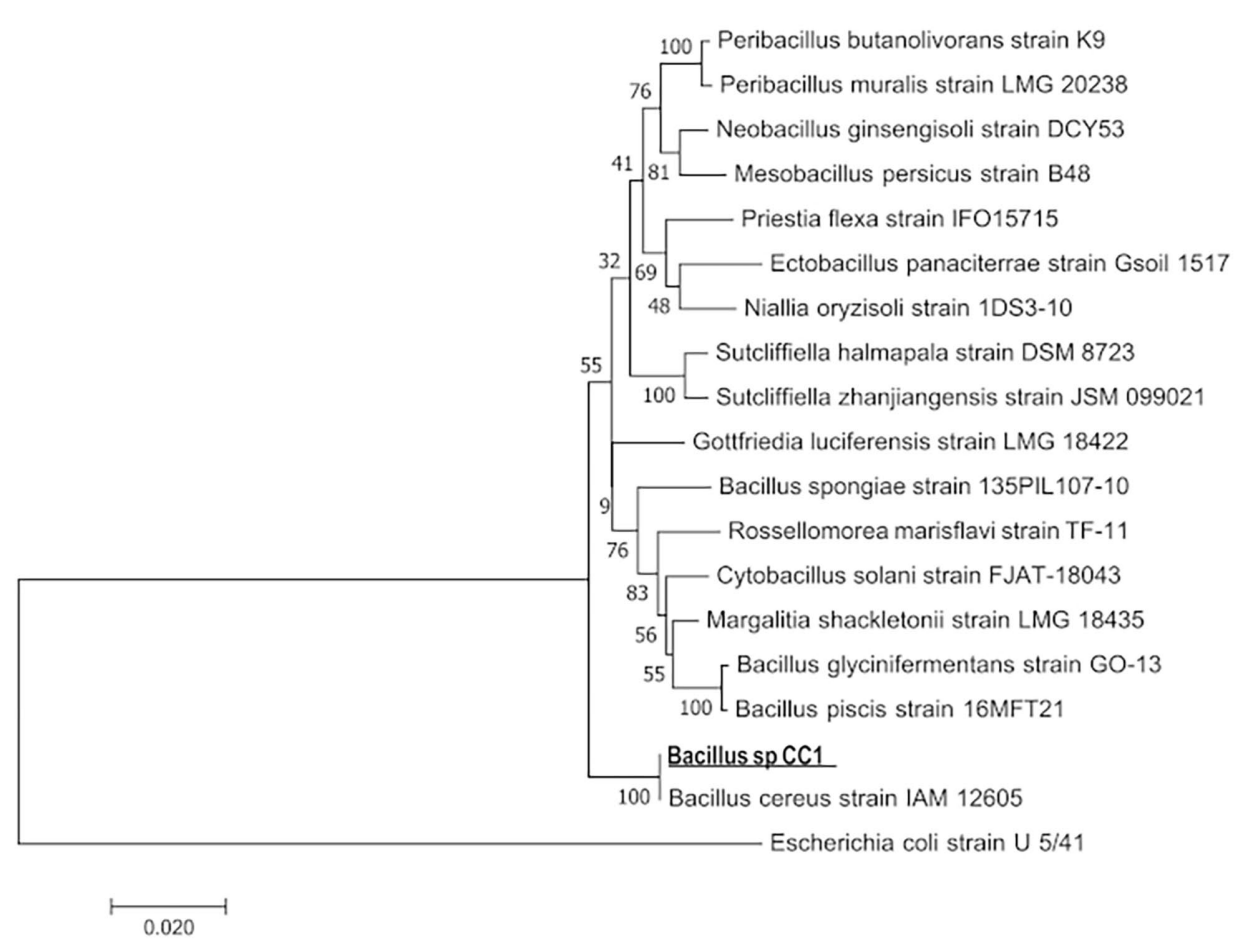
Phylogeny tree of the CC1 isolated strain prepared by MEGA software with the neighbour-joining method showing the phylogenetic similarity of the CC1 strain with *Bacillus cereus* strain IAM 12,605

**Fig. 5:**
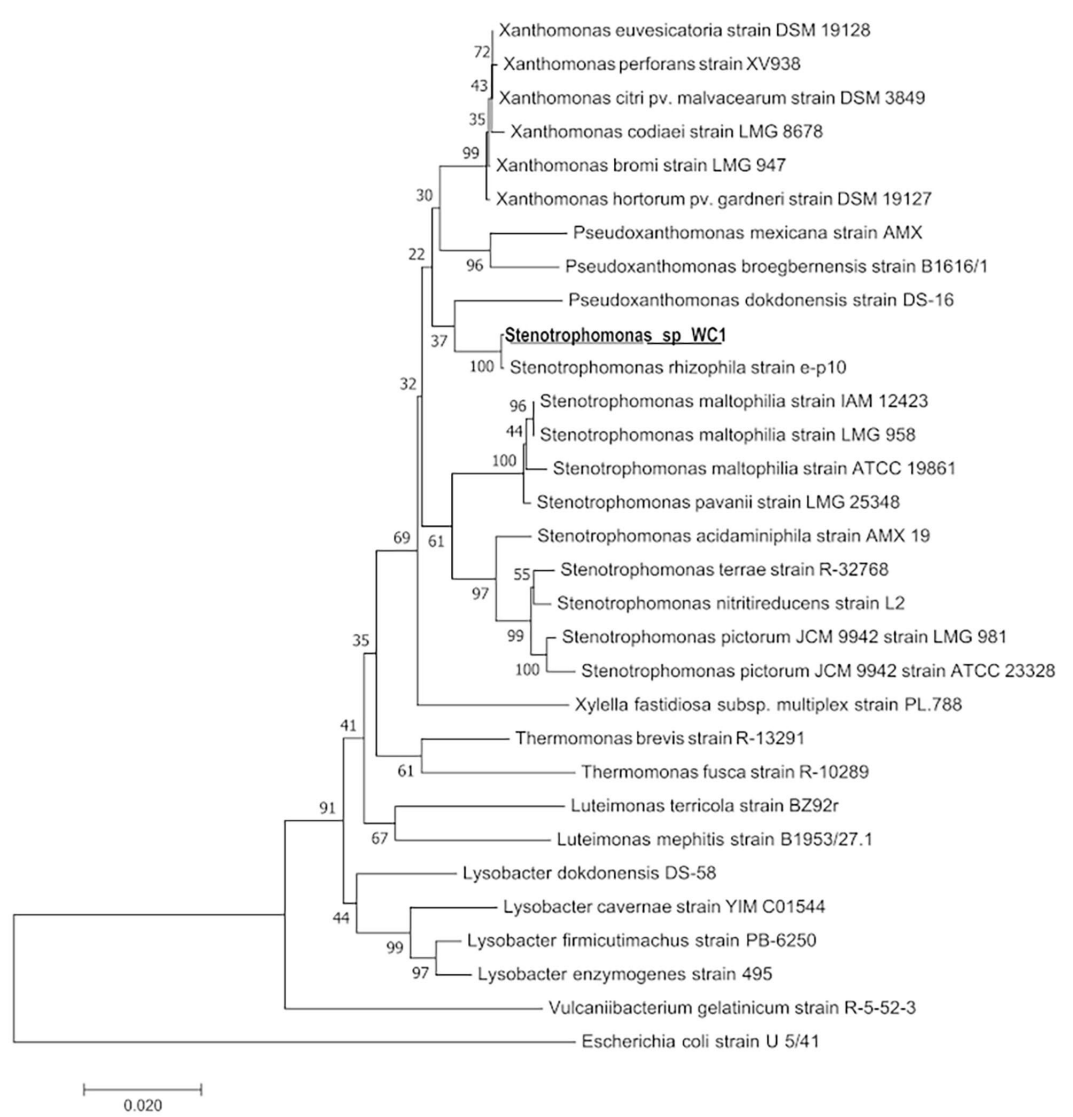
Phylogeny tree of the identification of the WC1 isolated strain prepared by MEGA software with the neighbour-joining method showing the phylogenetic similarity of the WC1 strain with *Stenotrophomonas rizophila* strain e-p10

The two isolated strains were molecularly identified by amplifying and sequencing the 16 S rDNA gene sequences, followed by comparison to a database containing known 16 S rDNA sequences. The results of this analysis showed that strain WC1 was closely related to *Stenotrophomonas rizophila* strain e-p10 with 99.8% sequence identity, while strain CC1 was 99.9% identical to *Bacillus cereus* strain IAM 12,605. Related sequences were registered with the Genbank under the accession numbers OR726222 and OR726221 respectively. The phylogenetic trees of these two isolated strains are illustrated in Figs. 4 and 5. In a prior study conducted by Li et al., it was documented that *Stenotrophomonas sp.* possesses the capability to degrade phenol in collaboration with *Advenella sp*. B9, even at concentrations as high as 1200 mg/litter. However, when acting independently, *Stenotrophomonas sp.* is only able to degrade phenol concentrations below 50 mg/L [33]. Similarly, Banerjee and his colleagues have also reported the ability to degrade phenol in two different strains of *Bacillus cereus* species, along with the metabolic pathway of phenol degradation in these isolates [34].

### Bilayer column experiment

**Fig. 6:**
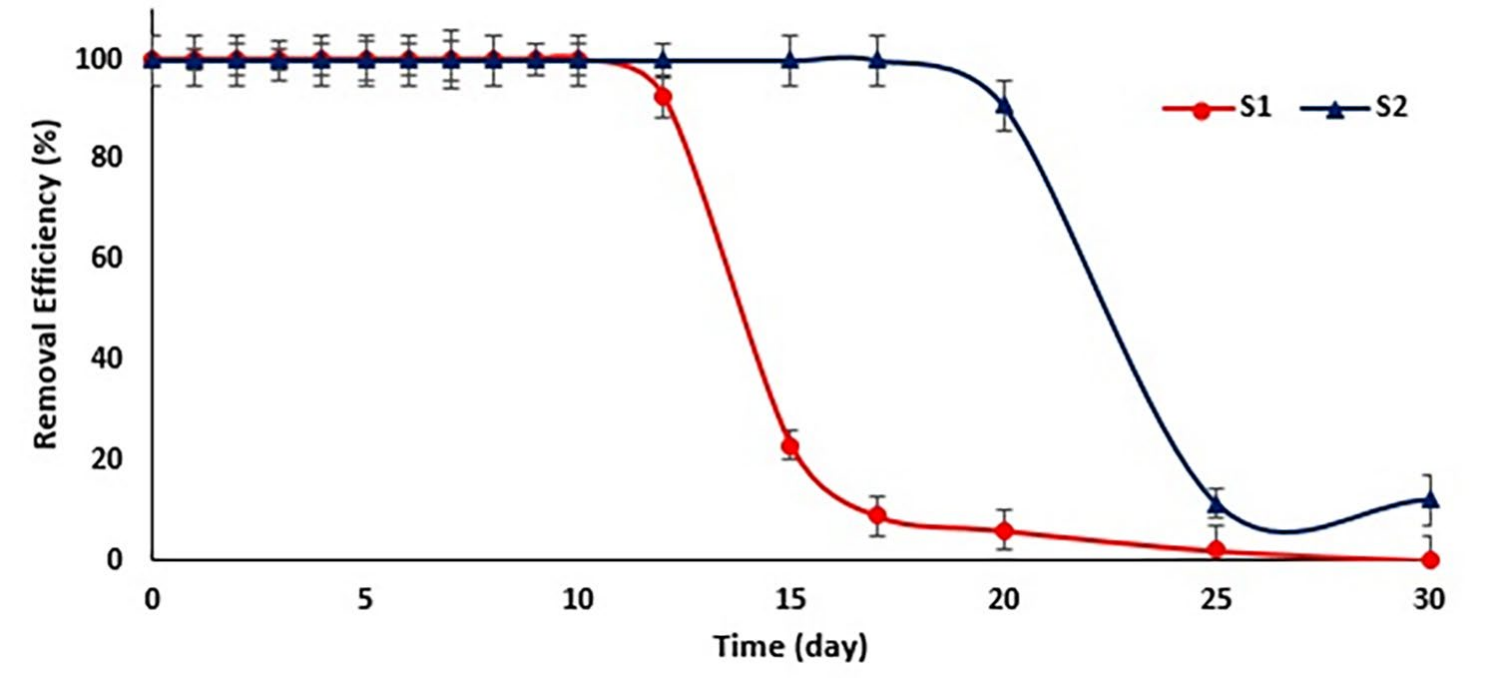
The percentage of phenol removal from the continuous flow of contaminated water from the output of column S1 (the column containing enzyme capsules) and the output of column S2, which shows the total removal by the first and second columns

Using two-layer systems is common in removing various pollutants, especially in systems where contaminants are removed by physical and chemical methods. For example, Malhotra and Suman used microbial laccases as efficient biocatalysts for the delignification and detoxification of lignocellulosic biomass, offering a green initiative for energy generation processes [35]. As previously stated, a series of columns were created to remove phenol from polluted waters through a synergistic approach. These columns consist of core-shell microcapsules with HRP enzyme (S1) and encapsulated degrading bacteria (S2). This system was operated twice: once using core-shell microcapsules containing crude extracted enzymes and another time using dialyzed enzymes. Figure 6 shows the removal efficiency of phenol by S1 and S2 columns when the S1 column was filled with the dialyzed enzyme core-shell microcapsules. As is clear from the diagram, the use of the enzyme column (S1) maintains the amount of phenol removal above 65% for 12 days alone, and then it decreases steeply and approaches zero. However, adding a microbial column (S2) increased the removal efficiency to above 95% for more than 20 days. After this period, phenol removal will decrease due to the loss of the HRP enzyme in the first column, probably because of a higher concentration of phenol input to the second column above bacterial tolerance. Similar results were obtained with lower removal efficiency for the system holding core-shell microcapsules containing crude extracted enzymes (the results are not shown). In research conducted by Gomez et al. in 2006, they used two stabilized HRP and SBP enzymes to remove phenol from polluted water flow in column reactors but used oxygen peroxide in the water flow to provide the necessary substrate for the oxidation and decomposition of phenol [36]. However, this research was the first to use HRP-containing core-shell microcapsules in the enzymatic pre-treatment of phenolic pollution before microbial degradation.

## Conclusion

The findings derived from this investigation, along with previous studies, suggest that phenol-degrading bacteria, particularly microflora found in groundwater, are vulnerable to elevated levels of phenol. These results suggest that the growth and activity of these microorganisms are completely inhibited and destroyed at excessive concentrations of phenol. Therefore, using microbial decomposition alone to biologically remove phenol from wastewater and water with high concentrations of phenol may not be sufficient due to the shock caused by the toxicity of phenol. Various pre-treatments, including enzyme treatment, can be helpful for this purpose. The results obtained in this study showed that the use of core-shell microcapsules containing HRP enzyme has a high ability to remove and reduce the concentration of phenol in polluted wastewater. This approach can be used as an excellent pre-treatment to prevent phenolic shock caused by the microbial decomposition method. However, considering the instability of the enzyme against phenol and hydrogen peroxide and the gradual loss of activity of microcapsules, it is necessary to renew the column containing microcapsules periodically. Nevertheless, the need to renew the enzyme column can be reduced by replacing soybean peroxidase instead of HRP, which is more stable against hydrogen peroxide, or by increasing the stability of HRP against hydrogen peroxide by using molecular mutations. Furthermore, enhancing the system’s effectiveness can be achieved through the careful selection of more robust decomposing strains, and gradually acclimatizing them to higher phenol concentrations, or introducing various synergistic strains. Furthermore, the system’s efficiency can be improved by employing a semi-continuous stream of hydrogen peroxide instead of calcium peroxide nanoparticles. Additionally, alternative composite materials can be utilized instead of alginate and chitosan to enhance the enzyme and microbial strains’ stability and effectiveness.

## Data Availability

All data are included in the manuscript and additional information, and further queries about sharing data can be directed to the corresponding author. The 16 SrRNA sequence of the *Stenotrophomonas* sp. WC1 was deposited into Genbank with accession #OR726222.1 https://www.ncbi.nlm.nih.gov/nuccore/OR726222.1, The 16 SrRNA sequence of the *Bacillus cereus* CC1 was deposited into Genbank with accession #OR726221.1 https://www.ncbi.nlm.nih.gov/nuccore/OR726221.
